# Can spheroid formation enhance mesenchymal stem cell homing in veterinary medicine? Insights and evidence gaps

**DOI:** 10.3389/fvets.2025.1622717

**Published:** 2025-09-24

**Authors:** Leonardo Martin, Guido R. Y. De Meyer

**Affiliations:** ^1^Laboratory of Physiopharmacology, Department of Pharmaceutical Sciences, University of Antwerp, Antwerp, Belgium; ^2^Center of Excellence Infla-Med, University of Antwerp, Antwerp, Belgium; ^3^Faculty of Pharmaceutical, Biomedical and Veterinary Sciences, University of Antwerp, Antwerp, Belgium

**Keywords:** mesenchymal stem cell, 3D culture, spheroid, stem cell homing, reverse translational research

## Introduction

Mesenchymal stem cells (MSCs) derived from adipose tissue have emerged as a promising tool in regenerative therapies across both human and veterinary medicine. A critical determinant of therapeutic success is the MSCs' ability to home to damaged or inflamed tissues. In human clinical and preclinical models, spheroid culture has been shown to augment the homing efficacy of MSCs by upregulating key chemokine receptors and enhancing cell survival (1, 27). Recent studies investigating MSC spheroids in canine models have begun to explore these effects in veterinary contexts (2, 3). A recent review presents the MSC homing cascade as a tightly regulated multistep process comprising tethering, rolling, activation, arrest, transmigration, and migration (4). While these mechanisms are well-characterized in human mesenchymal stem cells, they remain insufficiently studied in veterinary models. Additional insights from the literature highlight that optimizing microenvironmental cues within spheroid cultures can significantly enhance MSC functionality in regenerative applications (5, 6).

## Mechanisms of enhanced homing via spheroid culture

The formation of MSC spheroids recapitulates a physiologically relevant three-dimensional (3D) microenvironment, which has been shown to upregulate key molecular mediators involved in cell trafficking and homing. Notably, 3D spheroid architecture enhances the expression of C-X-C chemokine receptor type 4 (CXCR4), integrins, and matrix metalloproteinases (MMP-9 and MMP-13), molecules that play pivotal roles in transendothelial migration and extracellular matrix remodeling (7–9, 28).

These molecules converge functionally within the SDF-1/CXCR4 chemotactic axis, a canonical pathway directing MSC homing to sites of tissue injury. Spheroid culture also induces localized hypoxia due to limited oxygen diffusion, which in turn stabilizes hypoxia-inducible factor 1-alpha (HIF-1α). This transcription factor has been shown to transcriptionally activate genes such as CXCR4 and other adhesion receptors, thereby enhancing MSC migratory capacity and responsiveness to chemokine gradients (1, 10, 11).

Experimental evidence supports that MSCs cultured as spheroids exhibit improved transmigration across endothelial monolayers and greater directional migration toward SDF-1 gradients (1). In a canine study, Lee et al. (12) reported significant upregulation of HIF-1α in 3D-cultured adipose-derived MSCs. Although their investigation focused on immunomodulatory properties, the observed induction of HIF-1α is mechanistically relevant to homing, given its role in enhancing CXCR4 expression. This suggests that the hypoxic microenvironment within spheroids may potentiate MSC chemotaxis, although direct migration assays were not conducted in that study.

Parallel insights from murine hind limb ischemia models show that modifying MSCs to overexpress proangiogenic factors such as GM-CSF significantly enhances their contribution to neovascularization, increasing both capillary density and arteriogenesis (25). These findings support the broader notion that preconditioning or modification strategies, including spheroid formation, can potentiate MSC homing and functional integration into ischemic tissues.

In addition to receptor-mediated mechanisms, emerging hypotheses suggest that MSC spheroids may secrete bioactive vesicles and paracrine factors that contribute to homing efficiency. Specifically, spheroid culture has been associated with elevated exosome release, potentially driven by cytoskeletal tension and hypoxia. While these vesicles have been implicated in immunomodulation and tissue repair, their direct role in facilitating MSC homing via chemokine gradient formation remains to be experimentally validated (13). Furthermore, these processes remain poorly characterized in veterinary studies. Thus, while spheroid culture clearly enhances several homing-related molecular pathways, further *in vivo* tracking studies in companion animals are necessary to delineate the specific contributions of hypoxia-induced signaling, surface receptor modulation, and secretome-derived factors to MSC homing in a species-specific context.

## Application in canine studies

Current evidence on the use of 3D spheroid MSCs in veterinary medicine remains limited but promising. Among the few original studies available, Lee et al. (12) demonstrated that 3D-cultured canine adipose tissue-derived MSCs (cAT-MSCs) exhibit enhanced immunomodulatory activity compared to their 2D counterparts. Specifically, spheroid-conditioned media significantly downregulated the expression of pro-inflammatory cytokines (TNF-α, IL-1β, IL-6) and promoted M2 macrophage polarization in canine macrophages (DH82 cell line). Additionally, the same study reported upregulation of HIF-1α, TGF-β1, and COX-2, further supporting the notion that spheroid formation creates a functionally distinct secretome.

While these *in vitro* findings suggest that 3D spheroid architecture enhances therapeutic potential in canine MSCs, there is a lack of direct *in vivo* validation in veterinary disease models. Notably, many studies reporting improved outcomes using cAT-MSCs in canine osteoarthritis, spinal cord injury, or cancer [e.g., (14–16)] have employed conventional 2D-expanded cells rather than 3D spheroids. Thus, although extrapolation from human and rodent studies provides valuable insight, application of spheroid-based MSC therapy in canine clinical settings remains largely unexplored, representing a critical gap in translational research.

Given that adipose-derived MSCs represent the predominant and most clinically feasible stem cell source in veterinary practice, it is important to examine their unique significance and the growing body of evidence for their homing potential.

## Significance of adipose-derived MSCs in veterinary applications

Adipose tissue has become the most widely used and practical source of mesenchymal stem cells in veterinary regenerative medicine. Compared to bone marrow, adipose-derived MSCs (cAT-MSCs) offer the advantages of minimally invasive harvest, higher cell yield, and consistent proliferative and immunomodulatory potential, which have made them the predominant stem cell product in companion animal clinical trials. Their relevance extends beyond accessibility, as accumulating evidence indicates that cAT-MSCs possess robust therapeutic efficacy in conditions such as osteoarthritis, spinal cord injury, and cancer, where migratory and homing behavior is critical for tissue repair and modulation of local immune responses.

Several *in vivo* studies provide support for the therapeutic contribution of cAT-MSC homing. In canine spinal cord injury, repeated allogeneic transplantation of cAT-MSCs promoted long-term functional recovery, suggesting effective engraftment and migration to lesion sites (16). Earlier reports of cell delivery in acute disk disease also demonstrated clinical improvements consistent with tissue integration of transplanted cAT-MSCs (15). Similarly, in canine cancer studies, adipose-derived MSCs modified for therapeutic gene delivery have shown the capacity to localize within tumor microenvironments (14), reinforcing the concept that adipose-derived MSCs can actively home to inflammatory and pathological tissues *in vivo*.

Emerging mechanistic insights from 3D spheroid cultures of cAT-MSCs further highlight their potential to enhance homing. Lee et al. (12) reported that canine spheroid-cultured MSCs exhibited upregulation of HIF-1α, TGF-β1, and COX-2, pathways closely linked with chemotaxis and immunomodulation. The hypoxia-driven induction of HIF-1α is particularly relevant, as it stabilizes CXCR4 expression, a central receptor in the SDF-1/CXCR4 chemotactic axis, and thereby primes cAT-MSCs for directional migration toward sites of injury. Complementary findings by Ichikawa et al. (3), who co-cultured canine adipose-derived MSCs with hepatocytes and endothelial cells, demonstrated spheroid-based cross-talk that supported vascularization and tissue integration, underscoring the translational value of 3D culture in shaping homing-relevant signaling networks.

Collectively, these studies underscore the importance of adipose-derived MSCs as a clinically feasible and biologically potent cell source in veterinary medicine. They also indicate that while homing-related pathways have been indirectly evidenced in canine cancer studies, direct biodistribution and migration tracking studies of cAT-MSCs remain sparse. Addressing this gap with *in vivo* imaging and standardized homing assays will be essential to fully validate the therapeutic advantage of spheroid-based cAT-MSC preparations in veterinary clinical settings.

**Figure 1:**
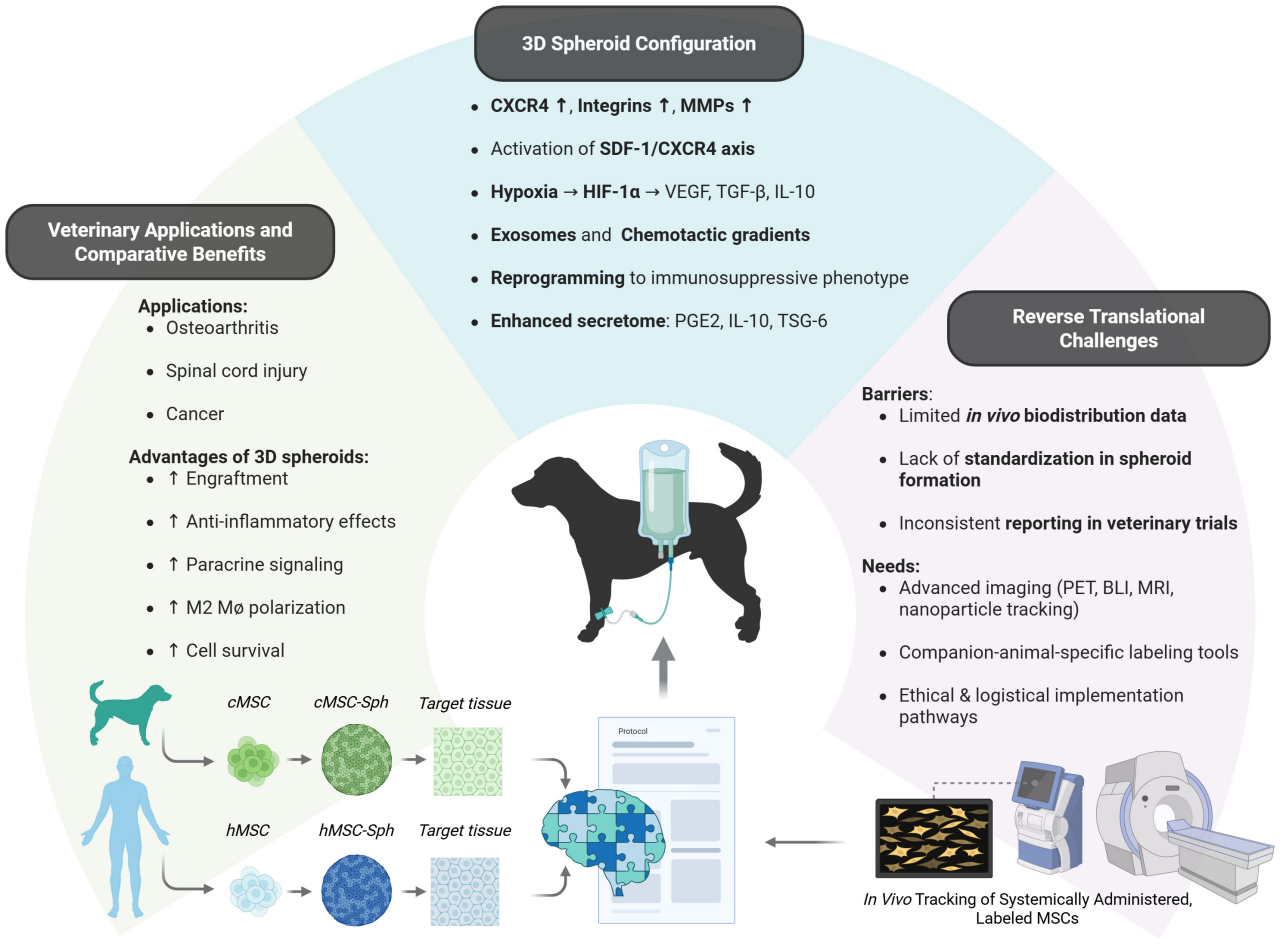
Mechanisms, advantages, and translational gaps of cAT-MSC spheroids in veterinary regenerative medicine. Three-dimensional culture of cAT-MSCs enhances homing via receptor activation, hypoxia-driven signaling, and secretome modulation. These mechanisms translate into improved outcomes in canine regenerative applications, particularly for inflammatory, orthopedic and neuro-regenerative disorders. However, challenges in standardization, *in vivo* tracking, and cross-species validation highlight the need for expanded reverse translational research. cMSC, canine mesenchymal stem cells; hMSC, human mesenchymal stem cells; cMSC-Sph, canine mesenchymal stem cell spheroids; hMSC-Sph, human mesenchymal stem cell spheroids. Created with BioRender.

These findings, when interpreted alongside advances in human MSC research, highlight the value of reverse translational approaches to refine spheroid-based protocols in veterinary systems. These mechanistic advantages, translational opportunities, and remaining evidence gaps are summarized in Figure 1.

## Reverse translational implications

The application of MSC spheroids in veterinary regenerative medicine is supported by a robust body of evidence derived from human preclinical and clinical studies. When cultured from human sources, MSCs cultured as 3D spheroids have consistently demonstrated enhanced therapeutic performance compared to monolayer-expanded cells. These enhancements include increased engraftment efficiency, prolonged *in vivo* survival, and amplified secretion of bioactive factors, such as vascular endothelial growth factor (VEGF), hepatocyte growth factor (HGF), and prostaglandin E2 (PGE2), all of which contribute to improved angiogenesis, immune modulation, and tissue repair (11, 17). The superior functionality of 3D spheroid MSCs is largely attributed to preserved cell–cell and cell–matrix interactions, hypoxia-induced signaling, and cytoskeletal reorganization within the spheroid microenvironment. These mechanistic insights offer a valuable framework for reverse translational application in veterinary medicine.

Despite this potential, several challenges limit the direct transfer of human protocols to veterinary practice. These include the lack of standardized methods for spheroid formation, size optimization, and preconditioning in animal systems, as well as limited *in vivo* data on biodistribution, persistence, and efficacy of spheroid MSC products in companion species (18). Moreover, inconsistencies in experimental design and outcome reporting across veterinary trials hinder cross-study comparisons and the establishment of best practices (19).

Lee et al. (12) underscore the importance of reproducible spheroid production methods and tailored delivery routes to maximize therapeutic efficacy in dogs. However, translating human protocols to veterinary settings is complicated by interspecies differences in MSC behavior, including distinct immunomodulatory pathways, receptor expression profiles, and secretome composition (20, 21). These discrepancies reinforce the need for species-specific investigations to validate spheroid-based therapies in non-human systems.

Rodent studies have also revealed that MSC therapeutic efficacy varies with the donor background, as demonstrated by differences between Balb/c- and C57BL/6-derived MSCs in hind limb ischemia studies (26). This variability underscores that intrinsic MSC properties, including tissue of origin, genetic background, and culture conditions, strongly influence their homing and therapeutic behavior. Extrapolating directly from human or murine systems to canine systems is therefore limited, reinforcing the need for species-specific validation in veterinary regenerative medicine.

To address the current limitations in understanding MSC homing and biodistribution in veterinary applications, *in vivo* imaging studies are critically needed. These studies can provide real-time insights into MSC migration dynamics, tissue-specific engraftment efficiency, and potential off-target effects following local or systemic administration. Advanced imaging technologies such as bioluminescence imaging (BLI), magnetic resonance imaging (MRI), positron emission tomography (PET), and nanoparticle-based labeling have greatly improved the capacity to track MSCs post-transplantation in human research (22–24). However, the application of these modalities in veterinary medicine is hindered by anatomical diversity, limited genetic labeling techniques, and a scarcity of species-specific molecular probes. Additionally, ethical considerations, resource constraints, and equipment accessibility further impede widespread implementation.

Establishing standardized protocols for spheroid generation, MSC labeling, administration, and post-delivery tracking will be essential to overcome these translational barriers. By adapting proven human methodologies to veterinary systems, it will be possible to achieve a more accurate characterization of MSC behavior *in vivo* and advance the clinical deployment of spheroid-based therapies in companion animals.

## Conclusion

The spheroid-based delivery of cAT-MSCs represents a promising strategy to enhance homing efficacy and therapeutic potency in regenerative veterinary medicine. Lessons drawn from human MSC research underscore the potential of applying 3D culture innovations in animal systems. Multiple studies have collectively emphasized the critical role of optimizing spheroid culture systems to improve mesenchymal stem cell retention, immunomodulatory potential, and functional integration within host tissues. However, translating these advantages into consistent veterinary outcomes necessitates further mechanistic investigations, especially into the stepwise homing cascade and its regulatory cues in non-human physiology. Bridging this knowledge gap will be essential for the successful clinical deployment of MSC spheroids in veterinary regenerative therapies.
